# Dataset on rbcL-based intra-specific diversity of *Gongronema latifolium* Benth: (Apocynaceae) in South-East Nigeria

**DOI:** 10.1016/j.dib.2022.107870

**Published:** 2022-01-26

**Authors:** Conrad Asotie Omonhinmin, Chinedu Charles Onuselogu, Enameguono Olomukoro

**Affiliations:** Department of Biological Sciences Biotechnology Cluster, College of Science and Technology, Covenant University, Canaan land Ota, Ogun State, Nigeria

**Keywords:** Evolution, Genetic diversity, *Gongronema latifolium*, Medicinal, Phylogeny, *rbcL* gene

## Abstract

*Gongronema latifolium* (Apocynaceae) is a versatile plant of nutritional and medicinal value and is widely distributed and endemic to the South-Eastern region of Nigeria. The plant is relatively wild and its natural habitat is threatened by deforestation, excessive exploitation and constant expansion of the urban areas into its endemic space. Hence, there is a need to understand its genetic diversity for breeding and conservation. The data consist of fourteen partial *rbcL* gene sequences, nucleotide compositions and amino acid profiles of *G. latifolium*. The data set provides insight on the species genetic diversity and evolution that is important for scientist and breeders alike as well as for conservation efforts of the species.

## Specifications Table

**Table:** 

Subject	Biological Science
Specific subject area	Agricultural, Genetic diversity, Phylogenetics, Evolution
Type of data	Tables, Figure
How data were acquired	Amplification of the *rbcL* gene through PCR and DNA Sanger sequencing.
Data format	Raw, Analyzed
Parameters for data collection	Whether the geographical locations of *G. latifolium* across South- East Nigeria affect intra-specific gene sequence variation in ribulose 1, 5 bisphosphate carboxylase/ oxygenase (*rbcL*)
Description of data collection	Young leaf samples of *G. latifolium* were collected in South- Eastern Nigeria (Anambra, Ebonyi, Imo, Enugu and Abia) Table 1. All accessions were evaluated using *rbcL* primers and the population diversity, nucleotide and amino acids compositions of the accessions were estimated using DnaSP 4.5. Codon usage bias and the codon usage indices were estimated using CodonW.
Data source location	The data locations are summarized in Table 1.
Data accessibility	The sequence data of the accessions have been deposited in NCBI GenBank data base sequence and has the following accession numbers; MH305573.1, MH305574.1, MH305578.1, MH305579.1, MH305580.1, MH305570.1, MH305571.1, MH305572.1, MH305581.1, MH305582.1, MH305583.1, MH305575.1, MH305576.1, MH305577.1. https://www.ncbi.nlm.nih.gov/nuccore/?term=Gongronema+latifolium

## Value of the Data

• This data provides information of the genetic diversity of *G. latifolium* sequences across South-Eastern Nigeria using information from partial *rbcL* gene sequences, nucleotide polymorphism and amino acids composition.
• The *rbcL* gene sequences can be employed by plant taxonomists to trace the molecular phylogeny, evolution and sub-speciation of *G. latifolium*.
• This data identifies areas of high genetic diversity of *G. latifolium* which can be adopted to create germplasm for species conservation.
• This data presents information on the amino acid composition and codon usage of the species.

## 1 Data Description

**Fig. 1:**
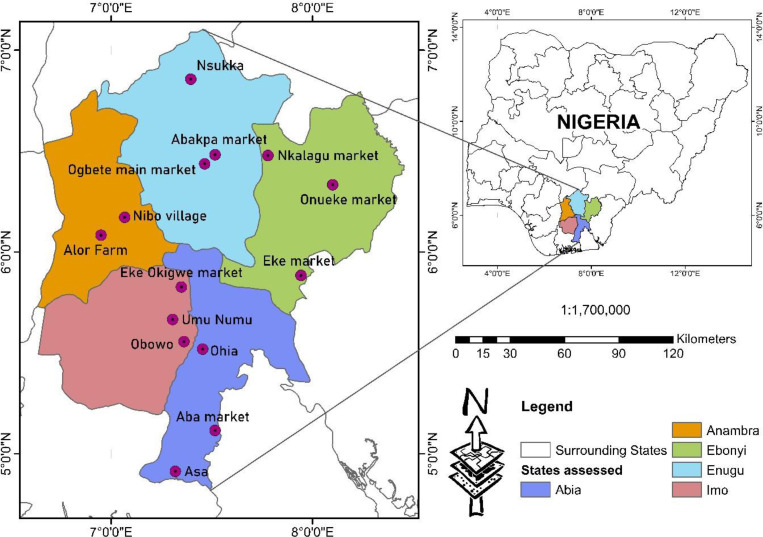
Species range and collection sites across South-East Nigeria.

*Gongronema latifolium* (Benth.) is classified in the family Apocynaceae and is of considerable nutritional and medicinal importance to the people of West Africa [1]. Despite its nutritional and medicinal uses, the plant is still relatively wild and repeatedly plagued by deforestation, excessive exploitation and constant expansion of the urban areas into rural areas as well as general poor land management practices in the distribution of *G. latifolium* [2]. The management and conservation of *G. latifolium* genetic resources across South-Eastern Nigeria is lacking, yet there is increased demand for its use for herbal formulations and as a leafy vegetable across the West African region and beyond, particularly with the spread of the Igbo cuisine across the region. Therefore, there is a need to understand the current genetic diversity of this species in Nigeria as well as to possibly create an active germplasm for the conservation and breeding of the species. The study presents the first *rbcL* gene sequences of *G. latifolium* from five Nigerian states. Table 1; lists the site collection details and the accessions of fourteen collections of *G. latifolium* as submitted to NCBI GenBank. Table 2; presents information about the accessions, including the % GC and the sequence length. Table 3, records the within collection area (state) genetic diversity of *G. latifolium*, which includes: number of segregating sites; within group mean distance; nucleotide diversity; and average number of nucleotide differences (k). Table 4, shows the amino acids and nucleotide compositions of the accessions of *G. latifolium*. Table 5, records the codon usage frequency table for *G. latifolium*. The genetic diversity of *G. latifolium* across the accessions is shown in Table 6. The codon usage indices of the accessions are represented in Table 7. Fig. 1 is the map of the collection sites across the study areas.

**Table 1:** Details on *Gongronema latifolium* accessions as submitted on NCBI GenBank and site collection information.

S/N	GenBank accession number ф	Locality	State	Altitude (m)	LGA	Latitude NS	Longitude EW	Herbarium number (vouchers)
1	MH305570.1	Aba market	Abia	205	Aba South	5° 6′ 55.8072″ N	7° 20′ 35.1852″ E	AbaCH001
2	MH305571.1	Ohia	Abia	97	Umuahia South	5° 31′ 6.708″ N	7° 27′ 17.64″ E	AbaCH002
3	MH305572.1	Asa	Abia	23	Ukwa West	4° 54′ 46″ N	7° 19′ 9″ E	AbaCH003
4	MH305573.1	Nibo village	Anambra	252	Awka South	06° 10′ 19N	7° 4′ 3E	AnaCH001
5	MH305574.1	Alor Farm	Anambra	160	Idemili South	6° 05′N	6° 57″E	AnaCH002
6	MH305575.1	Onueke market	Ebonyi	111	Ezza South	6°20′N	8°06′E	EboCH001
7	MH305576.1	Nkalagu	Ebonyi	126	Ishielu	6° 28′ 42″ N	7° 46′ 44″ E	EboCH002
8	MH305577.1	Eke market	Ebonyi	106	Afikpo	5° 53′ 2.5008″ N	7° 56′ 34.0008″ E	EboCH003
9	MH305578.1	Nsukka	Enugu	430	Nsukka	6° 51′24″ N	7°23′45″ E	EnuCH001
10	MH305579.1	Ogbete main market	Enugu	223	Enugu North	9° 2′44″ N	7° 27′ 54″ E	EnuCH002
11	MH305580.1	Abakpa market	Enugu	223	Enugu East	6° 28′ 56.2584″ N	7° 30′ 59.4468″ E	EnuCH003
12	MH305581.1	Obowo	Imo	213	Obowo	5° 33′ 21.0528″ N	7° 21′ 43.3476″ E	ImoCH001
13	MH305582.1	Umu Numu	Imo	252	Ehime-Mbano	5° 39′ 55.7784″ N	7° 18′ 20.646″ E	ImoCH002
14	MH305583.1	Eke Okigwe market	Imo	158	Okigwe	5° 49′ 35.1912″ N	7° 20′ 57.3612″ E	ImoCH003

*Voucher specimens in form of leaves and seed as herbarium specimens were deposited in the herbarium repository of the Department of Biological Sciences, Covenant University, Ota, Nigeria.

**Table 2:** Summary of the *rbcL* sequences of *G. latifolium* accessions.

Accession Number	State	% GC	Sequence Length
MH305570.1	Abia	44.10%	521
MH305571.1	Abia	44.60%	514
MH305572.1	Abia	44.20%	529
MH305573.1	Anambra	44.40%	532
MH305574.1	Anambra	44.60%	514
MH305575.1	Ebonyi	44.20%	523
MH305576.1	Ebonyi	44.70%	519
MH305577.1	Ebonyi	44.30%	519
MH305578.1	Enugu	44.40%	525
MH305579.1	Enugu	44.70%	514
MH305580.1	Enugu	44.60%	514
MH305581.1	Imo	44.10%	524
MH305582.1	Imo	44.60%	518
MH305583.1	Imo	44.30%	519

**Table 3:** Intra-specific diversity of *rbcL G. latifolium* accessions.

State	No. of accessions	No. of segregating sites	Within Group Mean Distance	Nucleotide Diversity	Average no. Nucleotide Differences k
Abia	3	9	0.00247	0.01167 ± 0.00550	6
Anambra	2	0	0	0	0
Ebonyi	3	9	0.00268	0.01258 ± 0.00593	6
Enugu	3	1	0.000265	0.00130 ± 0.00061	0.667
Imo	3	2	0.00053	0.00493 ± 0.00239	2.352

**Table 4:** Nucleotide and amino acid compositions for *G. latifolium* accessions.

Nucleotide/ Amino acid composition	MH305570.1	MH305571.1	MH305572.1	MH305573.1	MH305574.1	MH305575.1	MH305576.1	MH305577.1	MH305578.1	MH305579.1	MH305580.1	MH305581.1	MH305582.1	MH305583.1
T	27.64	27.64	27.85	27.64	27.64	27.64	27.64	27.64	27.64	27.64	27.64	27.64	27.64	27.85
C	22.78	22.78	22.78	22.78	22.78	23	22.78	22.78	22.78	22.78	22.78	22.78	22.78	22.57
A	27.64	27.64	27.85	27.64	27.64	27.85	27.64	27.64	27.64	27.64	27.64	27.64	27.64	27.64
G	21.94	21.94	21.52	21.94	21.94	21.52	21.94	21.94	21.94	21.94	21.94	21.94	21.94	21.94
Ala	8.23	8.23	7.59	8.23	8.23	7.59	8.23	8.23	8.23	8.23	8.23	8.23	8.23	8.23
Cys	1.27	1.27	1.27	1.27	1.27	1.27	1.27	1.27	1.27	1.27	1.27	1.27	1.27	1.27
Asp	5.06	5.06	5.06	5.06	5.06	5.06	5.06	5.06	5.06	5.06	5.06	5.06	5.06	5.06
Glu	7.59	7.59	7.59	7.59	7.59	7.59	7.59	7.59	7.59	7.59	7.59	7.59	7.59	7.59
Phe	3.8	3.8	3.8	3.8	3.8	3.8	3.8	3.8	3.8	3.8	3.8	3.8	3.8	3.8
Gly	8.86	8.86	8.86	8.86	8.86	8.86	8.86	8.86	8.86	8.86	8.86	8.86	8.86	8.86
His	0.63	0.63	1.27	0.63	0.63	1.27	0.63	0.63	0.63	0.63	0.63	0.63	0.63	0.63
Ile	5.06	5.06	5.06	5.06	5.06	5.06	5.06	5.06	5.06	5.06	5.06	5.06	5.06	5.06
Lys	5.06	5.06	5.06	5.06	5.06	5.06	5.06	5.06	5.06	5.06	5.06	5.06	5.06	5.06
Leu	9.49	9.49	9.49	9.49	9.49	9.49	9.49	9.49	9.49	9.49	9.49	9.49	9.49	9.49
Met	0.63	0.63	0.63	0.63	0.63	0.63	0.63	0.63	0.63	0.63	0.63	0.63	0.63	0.63
Asn	2.53	2.53	1.9	2.53	2.53	1.9	2.53	2.53	2.53	2.53	2.53	2.53	2.53	2.53
Pro	8.23	8.23	8.23	8.23	8.23	8.23	8.23	8.23	8.23	8.23	8.23	8.23	8.23	8.23
Gln	2.53	2.53	2.53	2.53	2.53	2.53	2.53	2.53	2.53	2.53	2.53	2.53	2.53	2.53
Arg	5.06	5.06	5.06	5.06	5.06	5.06	5.06	5.06	5.06	5.06	5.06	5.06	5.06	5.06
Ser	3.8	3.8	3.8	3.8	3.8	3.8	3.8	3.8	3.8	3.8	3.8	3.8	3.8	3.8
Thr	8.86	8.86	9.49	8.86	8.86	9.49	8.86	8.86	8.86	8.86	8.86	8.86	8.86	8.86
Val	6.33	6.33	6.33	6.33	6.33	6.33	6.33	6.33	6.33	6.33	6.33	6.33	6.33	6.33
Trp	1.27	1.27	1.27	1.27	1.27	1.27	1.27	1.27	1.27	1.27	1.27	1.27	1.27	1.27
Tyr	5.7	5.7	5.7	5.7	5.7	5.7	5.7	5.7	5.7	5.7	5.7	5.7	5.7	5.7

**Table 5:** Codon Usage of *G. latifolium* accessions.

Codon	Count	RSCU*	Codon	Count	RSCU	Codon	Count	RSCU	Codon	Count	RSCU
**UUU(F)**	3	1	UCU(S)	3	3	UAU(Y)	5	1.11	UGU(C)	1	1
**UUC(F)**	3	1	UCC(S)	1	1	UAC(Y)	4	0.89	UGC(C)	1	1
**UUA(L)**	2	0.8	UCA(S)	1	1	UAA(*)	0	0	UGA(*)	0	0
**UUG(L)**	5.1	2.06	UCG(S)	0	0	UAG(*)	0	0	UGG(W)	2	1
**CUU(L)**	4	1.6	CCU(P)	5.8	1.78	CAU(H)	1.1	2	CGU(R)	3	2.25
**CUC(L)**	0	0	CCC(P)	3.2	0.99	CAC(H)	0	0	CGC(R)	1	0.75
**CUA(L)**	2	0.8	CCA(P)	3	0.92	CAA(Q)	3	1.5	CGA(R)	3	2.25
**CUG(L)**	1.9	0.74	CCG(P)	1	0.31	CAG(Q)	1	0.5	CGG(R)	0	0
**AUU(I)**	4	1.5	ACU(T)	7.1	2.02	AAU(N)	1.1	0.56	AGU(S)	0	0
**AUC(I)**	4	1.5	ACC(T)	3	0.85	AAC(N)	2.8	1.44	AGC(S)	1	1
**AUA(I)**	0	0	ACA(T)	3	0.85	AAA(K)	8	2	AGA(R)	1	0.75
**AUG(M)**	1	1	ACG(T)	1	0.28	AAG(K)	0	0	AGG(R)	0	0
**GUU(V)**	5	2	GCU(A)	5.9	1.82	GAU(D)	7	1.75	GGU(G)	4	1.14
**GUC(V)**	0	0	GCC(A)	3	0.93	GAC(D)	1	0.25	GGC(G)	2	0.57
**GUA(V)**	5	2	GCA(A)	3	0.93	GAA(E)	10	1.67	GGA(G)	4.1	1.18
**GUG(V)**	0	0	GCG(A)	1	0.31	GAG(E)	2	0.33	GGG(G)	3.9	1.1

*RSCU: Relatively synonymous codon usage.

**Table 6:** Genetic diversity of fourteen *G. latifolium* accessions.

Index	Value
Number of haplotypes	4
Haplotype diversity	0.396 ± 0.159
Nucleotide diversity	0.00493 ±0.00239
Average no. Nucleotide Differences (k)	2.352
No. of segregating sites	9

**Table 7:** Codon usage indices per accession.

	Codon Usage Parameters											
Accessions	T3s	C3s	A3s	G3s	CAI	CBI	Fop	Nc	GC3s	GC	L_sym	L_aa
**MH305570.1**	0.4755	0.2168	0.375	0.1562	0.267	0.136	0.494	51.38	0.3	0.439	170	173
**MH305571.1**	0.2636	0.2713	0.304	0.4052	0.141	-0.057	0.338	61	0.532	0.451	154	165
**MH305572.1**	0.4658	0.2329	0.3768	0.1462	0.262	0.14	0.497	50.02	0.306	0.441	173	176
**MH305573.1**	0.4795	0.2192	0.3669	0.1603	0.273	0.144	0.5	50.69	0.305	0.443	174	177
**MH305574.1**	0.2636	0.2713	0.304	0.4052	0.141	-0.057	0.338	61	0.532	0.451	154	165
**MH305575.1**	0.4437	0.2465	0.3957	0.1374	0.258	0.118	0.485	49.9	0.31	0.441	171	174
**MH305576.1**	0.2595	0.2672	0.2992	0.4153	0.142	-0.061	0.333	61	0.538	0.453	156	167
**MH305577.1**	0.3116	0.3478	0.3423	0.1881	0.136	0.018	0.426	43.27	0.453	0.465	148	159
**MH305578.1**	0.2652	0.2652	0.2992	0.4153	0.141	-0.066	0.331	61	0.535	0.45	157	168
**MH305579.1**	0.2615	0.2692	0.3016	0.4103	0.14	-0.062	0.335	61	0.535	0.453	155	165
**MH305580.1**	0.2636	0.2713	0.304	0.4052	0.141	-0.057	0.338	61	0.532	0.451	154	165
**MH305581.1**	0.4722	0.2222	0.375	0.1562	0.269	0.14	0.497	51.45	0.304	0.439	171	174
**MH305582.1**	0.2615	0.2692	0.3016	0.4103	0.141	-0.059	0.335	61	0.535	0.452	155	166

## 2 Experimental Design, Materials and Methods

### 2.1 Plant material

Specimens of *Gongronema latifolium* were collected in South-Eastern Nigeria, of five states; (Anambra, Abia, Imo, Enugu, and Ebonyi) (Fig. 1). The fresh leaf samples of the accessions were silica gel dried in labelled air-tight bags, and held at −80° prior to molecular analysis at the Bioscience Laboratory, International Institute of Tropical Agriculture (IITA), Ibadan Nigeria.

### 2.2 Genomic DNA extraction

Genomic DNA was extracted using the CTAB protocol [3].

### 2.3 Gene amplification and DNA sequencing

A portion of the chloroplast ribulose 1, 5-bisphosphate carboxylase (*rbcL*) gene was amplified with the rbcL-F (ATGTCACCACAAACAGAGACTAAAGC) and rbcL-R (GTAAAATCAAGTCCACCRCG) primers [4]. The PCR amplicon were sequenced at Inqaba biotechnical Industries (Pty) Ltd, South Africa.

### 2.4 Data analysis

Sequences were aligned using the Geneious Basic [5] with default settings to obtain the % GC and sequence lengths.

Population diversity indices such as numbers of segregating sites (S), haplotype number (h), haplotype diversity (Hd), nucleotide diversity (π) and average number of pairwise nucleotide differences within the population (K), were estimated using DnaSP 4.5 [6].

The nucleotide and amino acid compositions and the codon usage frequency table of *G. latifolium* were estimated using DnaSP 4.5.

Codon usage indices were calculated using CodonW as implemented on a public Galaxy server (https://galaxy.pasteur.fr/).

## CRedit Author Statement

**Conrad Asotie Omonhinmin:** Conceptualization, Methodology, Supervision; **Chinedu Charles Onuselogu:** Writing – review & editing, Writing – original draft preparation; **Enameguono Olomukoro:** Sequences submission on GenBank.

## Declaration of Competing Interest

The authors declare that they have no known competing financial interests.
